# Patient and public involvement in health and social care doctoral research: recommendations co-produced by doctoral researchers, public contributors, and a public involvement coordinator

**DOI:** 10.1186/s40900-026-00887-4

**Published:** 2026-06-18

**Authors:** Jordan Curry, Sarah P Bowers, Alexandra Wray, Anne Fearfull, Helen Roberts

**Affiliations:** 1 Wolfson Palliative Care Research Centre, Hull York Medical School, University of Hull, Allam Medical Building 3rd Floor, Cottingham Road, Hull, East Yorkshire, Hull, HU6 7RX UK; 2 The Activity and Nutrition in Cancer Research Group, Hull York Medical School, University of Hull, Hull, UK; 3 School of Medicine, University of St Andrews, St Andrews, UK; 4 York St John University, Lord Mayor’s Walk, York, YO31 7EX UK; 5 Hull York Medical School, University of Hull, Allam Medical Building 3rd Floor, Cottingham Road, Hull, East Yorkshire, HU6 7RX Hull, UK

**Keywords:** Patient and public involvement (PPI), Public contributors, PhD research, Doctoral studies, Research co-production, Public engagement, Collaborative research, Participatory research, PPI in doctoral training, Reflections on involvement

## Abstract

**Background:**

Patient and Public Involvement (PPI) is a continually growing component of health and social care research in the UK and internationally, ensuring that research is conducted with and by the public rather than simply about them. Doctoral research represents a critical opportunity to embed meaningful PPI early in researchers’ careers, yet practical recommendations tailored to this context remain limited. Existing studies often focus on individual case reflections or programme-level evaluations, with few capturing the collective experiences of doctoral researchers, PPI contributors, and coordinators. This paper aims to co-produce practical recommendations to support meaningful patient and public involvement in doctoral health and social care research.

**Main body:**

This paper presents co-produced reflections and recommendations to support PPI in doctoral research. The writing process was approached collaboratively, with a team incorporating public contributors, doctoral researchers, and a public involvement coordinator working together through iterative discussion and revision. The approach aligns with the principles of authentic co-production, creating both “space to talk” through open, respectful dialogue and “space to change” through mutual adaptation. The team’s experiences highlighted both shared benefits and challenges within the process. PPI enriched doctoral research by refining study design, questions, enhancing recruitment, and improving accessibility of outputs, while also offering PPI contributors opportunities for learning and impact. Challenges included managing emotional topics, balancing timelines, and recognising that not all suggestions could be implemented. The coordinator’s role proved vital in bridging between researchers and public contributors, supporting communication, reimbursement processes, and continuity of relationships. Doctoral researchers emphasised the importance of building trust, using accessible language, and developing distinct relational skills alongside academic expertise. From these collective insights, the team developed practical recommendations for three key audiences: doctoral students, PPI contributors, and PPI coordinators.

**Conclusion:**

PPI in doctoral research offers mutual benefits for contributors and early-career researchers, strengthening both research quality and personal and professional development. Genuine co-production requires time, respect, and institutional support, but can foster strong partnerships and more relevant, impactful research. The recommendations presented here aim to help doctoral researchers, PPI contributors, and coordinators embed authentic, equitable, and sustainable involvement across health and social care research.

## Abstract (plain language summary)

Patient and Public Involvement (PPI) means that research is carried out with and by members of the public, rather than to or about them. It helps research to be more relevant, inclusive, and useful for the people it aims to benefit. This paper describes and examines the experience of involving patients and the public in doctoral (PhD) research. Doctoral projects often have limited funding and time, but they also offer opportunities for close, long-term collaboration between students and public contributors. The aims and content of the paper was developed collaboratively by doctoral researchers, public contributors, and a PPI coordinator who reflected together on what worked well and what could be improved. The process showed the importance of building trust, communicating clearly, and recognising that both researchers and public contributors bring valuable skills and knowledge. Having a PPI coordinator to support students and contributors, provide training, and manage practical arrangements such as payments was also found to be helpful. From these shared experiences, practical recommendations were developed for doctoral students, public contributors, and PPI coordinators. These focus on getting started, building relationships, communicating effectively, and ending involvement respectfully. Meaningful PPI during doctoral research was found to improve study quality and impact for patients, families, and the wider public, while helping new researchers to develop collaborative and inclusive ways of working that keep the focus on what matters to patients and the public.

## Background

Patient and Public Involvement (PPI) is an established and growing component of health and social care research in the UK [1] and countries such as Australia, the USA, and Canada [2]. However, limited understanding and, at times, misunderstandings of its nature and role remain common. PPI refers to research carried out with or by members of the public, rather than to, about, or for them [3]. Involving patients and the public across research design, delivery, and dissemination is increasingly recognised as essential for ensuring that research is relevant, inclusive, and impactful [4, 5].

Doctoral research presents a particularly important setting for PPI. Doctoral projects not only contribute to the evidence base but also shape the professional development of early career researchers. Meaningful involvement during this formative stage can help align research with real-world needs, enhance study design, strengthen recruitment strategies, and ensure outputs are accessible and impactful [6–8]. Public contributors also report personal benefits, including the opportunity to share expertise, build skills, and contribute to positive change [9, 10].

In the UK, the National Institute for Health and Care Research (NIHR) has played a central role in promoting and embedding public involvement in research, from the late 1990s onwards. NIHR guidance emphasises embedding PPI throughout the research cycle, and this expectation extends to doctoral studies. For doctoral researchers, this presents both opportunities and challenges. Projects often operate with limited budgets and resources, strict timelines, and steep learning curves in both academic and engagement skills. At the same time, doctoral research offers unique conditions for sustained partnership: long project durations, opportunities for co-learning, and the chance to embed good practice early in a researcher’s career [6, 10].

The existing literature highlights both the benefits and challenges of PPI in doctoral research. Benefits include enhanced study design, improved cultural sensitivity and accessibility, and more diverse and effective dissemination outputs [6–8]. Reported challenges include balancing PPI with research timelines (particularly fixed doctoral funding and submission deadlines), addressing power imbalances, ensuring fair recognition and reimbursement, and managing ethical or emotional complexities, particularly in sensitive contexts such as paediatrics and bereavement [8]. Without institutional support, PPI can risk becoming tokenistic, with contributors excluded from later stages such as data analysis or authorship, representing a missed opportunity for genuine co-production [4]. Engaging PPI contributors from the outset encourages genuine co-production of the research process, for example, in qualitative work where early input may help ensure that questions and language are appropriately framed.

Several doctoral exemplars provide insights into how these challenges can be navigated. Advisory panels can offer structured, ongoing involvement [10]; “research buddy” models can foster one-to-one relationships that sustain patient perspectives throughout [9]; and long-term co-production within PhD programmes can reduce student isolation, build mutual learning, and improve the relevance and quality of outputs [6]. Yet most published accounts are either single-case reflections [7, 8], student-authored experiences [5], or programme-level evaluations focused on infrastructure [4, 10]. Few studies bring together the perspectives of doctoral researchers, public contributors, and coordinators to generate collective, transferable guidance.

To address this gap, our paper presents recommendations co-produced by a group of three doctoral researchers across two research programmes at two universities, alongside PPI contributors and a PPI coordinator. Two of the doctoral researchers (JC and AW) were based at the same university, supported by a PPI coordinator (HR), with PPI contributors (CS and AS) involved in their doctoral projects. The third doctoral researcher (SPB) was based at a different university and worked with a separate PPI contributor (AF). All worked within the field of palliative care research, including end-of-life and bereavement. In the spirit of “Nothing About Me Without Me” [11], we share lessons learned across these distinct doctoral projects, drawing on experiences where PPI was initiated early and shaped by both funder expectations and student-led motivation. We reflect on opportunities, challenges, and enablers of effective PPI from the perspectives of doctoral researchers, PPI contributors, and coordination support and aim to provide clear, practical recommendations tailored to three key groups: (1) doctoral researchers, (2) PPI contributors, and (3) PPI coordinators, to support meaningful and sustainable PPI in doctoral research in health and social care.

## Main body

### Our approach

The process of writing this paper was approached openly and collaboratively. Throughout, we aimed to create an environment that valued the perspectives and expertise of public contributors, researchers, and the public involvement coordinator. There were multiple overlapping ways in which we had worked together previously, and these relationships enabled us to take a conversational and flexible approach to sharing our experiences and writing this paper. In reflecting on this process, it was recognised that the approach taken aligns with the principles of authentic co-production described by Knowles et al., (2021), particularly the importance of creating both *space to talk*, open, respectful dialogue, and *space to change*, a willingness to adapt ideas and processes in response to one another [12].

A public involvement coordinator was a member of the team. Such a perspective is often missing in the literature about public involvement, either because the role itself does not exist within a team or research centre, or because the contribution of that role to the work is invisible [13].

At the beginning of the process, we held an introductory meeting to share our motivations and agree on a shared understanding of our purpose and approach. We used mixed methods to gather and understand our experiences and co-design our recommendations. An online survey (via Qualtrics) enabled each team member to respond to a series of open questions and share initial reflections based on their own experience. These contributions were explored collectively through a series of online meetings that acted as co-design spaces, supporting iterative discussion and shared sense-making. Each session focused on a different perspective (public contributors, researchers, and public involvement coordinator).

The researchers and the public involvement coordinator shared responsibility for organising and hosting these sessions, with different members chairing sessions at different time points. Our prior relationships and experiences of hosting, facilitating, and taking part in public involvement conversations enabled everyone to speak freely and challenge where necessary. The meetings lasted as follows: Introductory meeting: 80 min; Workshop 1: 55 min; Workshop 2: 60 min; and Workshop 3: 85 min. Following these sessions, three additional 60-minute meetings were held to support the iterative development of the manuscript and to review the evolving recommendations.

Over a year-long process, there was a shared wish among the public co-authors to produce a practical and accessible resource for doctoral students and future PPI collaborators, rather than a purely academic paper. The recommendations (*see* Sect.  2.3*Recommendations*) are intended as a first step toward that goal and reflect an ongoing effort to make collaboration between doctoral researchers and PPI contributors smoother, more equitable, and grounded in mutual learning.

### Our experiences and reflections

#### Experiences of PPI from PPI contributors

The PPI contributors brought a range of lived experiences to the doctoral research projects they supported, drawing on both personal perspectives and their professional or voluntary backgrounds.

The PPI contributors had a range of reasons for getting involved in the doctoral research projects. Many described past experiences of loved ones’ palliative care, death and bereavement that had been negative and wanted to create something positive out of difficult experiences. The relevance of the research topics to their own lives also encouraged participation, especially when presented in a way that felt accessible and meaningful. Being part of the projects gave those involved the chance to be a voice for others.

It was important to all contributors to feel engaged and involved, to know that their input was having an impact on the doctoral research.

Some had been part of other projects, both previously and subsequently, where their involvement had felt tokenistic, and the impact of their involvement was less clear. Being kept up to date with each stage of the project, including knowing about setbacks and challenges, was seen as important. Otherwise, PPI contributors described feeling frustrated at not knowing what was happening with the study. Those involved did not expect all of their suggestions or ideas to always be acted upon. Instead, knowing that their input was properly considered was seen as far more important:

*Engagement for me means getting useful feedback and feeling fully involved*,* not just an accessory. (PPI contributor)*

Delivering presentations, e.g. at conferences, jointly alongside the doctoral researchers was one way that contributors felt given the opportunity to be directly involved in sharing the messages arising from the research.

Being part of a wider PPI group was seen as positive to the overall experiences of participating in research projects. Working alongside people with similar experiences was seen as a positive, particularly when knowing such experiences were valued and considered:

*Working with like-minded people is always enjoyable*,* seeing that people’s experiences are sought and taken seriously. (PPI contributor)*

It was acknowledged that the topics of the research, palliative care, death and bereavement, could make some discussions emotional and challenging, but knowing others in the group were non-judgemental and empathetic eased such conversations. Inclusive groups, where individuality was respected, and contributors could be true to their roots, were seen as key to facilitating joint learning from collective experiences. PPI can take many forms, and in some contexts, it requires particularly sensitive or nuanced approaches, especially when involving people who may be emotionally vulnerable or dealing with profound experiences.

Working with a PhD student researcher was seen as positive by all the PPI contributors. Some had previously completed their own PhDs and remembered the challenges that these bring. Whilst their role as a PPI contributor was not to provide supervision to the doctoral students, they did feel that past experience equipped them with the ability to empathise with the trials and tribulations that doctoral research brings. A protectiveness and vested interest in seeing the PhD student develop and be successful in their research and degree was felt by the PPI contributors. They described working with PhD students as a particularly flexible and transparent process, compared to their experiences with more established academics:

*It felt “freer” than working with an established academic. (PPI contributor)*

Knowing that the individual doctoral researcher was leading the research project made communication and relationship building easier compared to working with large, established research teams. However, contributors did feel that some initial training on how best to work alongside researchers, particularly doctoral researchers, would have been beneficial to allow them to feel comfortable in the group, know their role and how best to input into doctoral research, especially as there is an awareness that doctoral research is time critical.

#### Experiences from a PPI coordinator

The PPI coordinator led the public involvement for a large team of researchers on a cancer research programme, including nine PhD students, working on multiple projects along the cancer journey, from cancer screening to palliative care.

Many of the researchers were new to public involvement in research, as were the PPI contributors and the PPI coordinator, who came from an NHS patient experience background. For the coordinator, this encouraged a flexible and open-minded approach to PPI, allowing different approaches for different projects, and a shared understanding of PPI as a learning process:

*We did not come into this space with fixed ideas or methods*,* and we had no infrastructure in place for public involvement in the beginning. (PPI coordinator)*

The coordinator identified some apprehension among researchers about the more flexible governance and methods used for public involvement as compared to research. Much of their experience in supporting PhD students was about unpicking the difference between academic skills and skills for public involvement. The use of Plain English is a particular example of a skill that underpins effective public involvement but runs counter to academic modes of communication. The learning curve to undertake robust public involvement was steep for some PhD students, and the coordinator had a key role in supporting this:

*My role was often to encourage them to ‘unlearn’ what they were learning as an academic*,* and this was not easy. (PPI coordinator)*

From the coordinator’s perspective, it was imperative that the skills and knowledge of PPI contributors were recognised and valued in their own right. Engaging meaningfully in PPI required researchers to develop skills in facilitating inclusive discussions and working collaboratively with people who bring lived experience rather than academic expertise. The public coordinator played a key role in ensuring PPI was seen as a process through which researchers could test ideas and methods, and receive feedback, advice, and reassurance from those with lived experience of their research topic.

It was also highlighted that it is still uncommon for research institutions to have a full-time, dedicated member of staff whose role is to support and advise PhD students in undertaking public involvement. Having this role embedded in a research programme had benefits for both PhD students and the individuals involved. The PPI coordinator was able to be a consistent point of contact and support for public contributors across and outside projects. This gave the chance to build relationships, develop trust and have an ongoing dialogue, ensuring that subsequently:

*People did not feel used or dropped at the end of projects. (PPI coordinator)*

Additionally, the PPI coordinator was able to support PhD students through co-facilitating meetings, feedback on how to write and present clear information, access to a pool of public contributors, access to a growing collection of learning resources and examples of good practice, and an established payment process for public contributors. Towards the end of the research programme, a comic book was created with a graphic artist [14] to capture the learning from embedding PPI and the research of two co-authors (AW and JC) feature in this (see appendix 7). Building and maintaining this bridge between the researchers and our public contributors, enabled a better understanding of the concerns, expectations and pressures faced.

#### Experiences of PPI from PhD students

The three doctoral researchers (JC, SPB, AW) had a range of experiences in involving PPI contributors, but a shared theme was that PPI enhanced their PhD experience and the research projects.

From the onset, the motivation to involve PPI was high amongst the three students. Their doctoral projects were all within the fields of survivorship, palliative, and end-of-life care, including the creation of a digital health platform (ExerciseGuide UK) [15, 16], the exploration of healthcare use in people with multiple long-term conditions at the end of life, and an investigation of bereavement support for children and surviving parents. Each researcher had a clinical background: a medical doctor, a clinical exercise physiologist, and a nurse, which provided them with familiarity in working alongside patients and the public. Yet they were conscious of the limits of their own professional and academic perspectives, recognising that PPI offered insights rooted in lived experience. As one noted:

*Although I had studied cancer in an academic sense*,* I lacked firsthand experience or insight into what it’s like to live with the disease.* (*PhD researcher*)

There was a strong commitment to ensure PPI was meaningful and to avoid it being tokenistic. PPI contributors were recruited through public involvement networks, advisory panels, patient organisations, social media, and community networks, with early meetings used to shape research questions and study design. In some cases, involvement directly redirected the research. For example, one student found that caregivers’ experiences of healthcare had been overlooked and refocused her study accordingly:

*I discussed with the PPI members how best to recruit patients to my study*,* however*,* after hearing their experiences*,* I realised that caregivers voices are often not heard*,* and so my research focus changed. (PhD researcher)*

At times, PPI perspectives challenged expectations held by both doctoral and senior researchers. One student conducted a Think-Aloud Usability Study with participants living with lung cancer, proposing website changes based on the analysis. Yet the PPI group disagreed with over half of these proposals. Instead of dismissing that, the student worked collaboratively with PPI to revise the platform, making it more accessible, user-centred, and impactful. While exposing work to critique after months of effort was daunting, however, they acknowledged that such challenges deepened their understanding and strengthened their outputs. PPI contributors highlighted issues of design, language, and sensitivity that researchers and even supervisors had not considered.

Given the sensitive nature of palliative care research, students reflected on the emotional labour carried by PPI contributors, many of whom revisited painful experiences to improve care for others. Building trust was therefore essential. At the suggestion of her PPI contributors, one doctoral researcher developed “get to know me” sessions for bereaved children, which eased anxiety, fostered rapport, and created a sense of shared purpose. Supporting contributors also required regular check-ins to manage emotional impact and to show contributors how their input had shaped the work.

Some programmes included PPI training for their doctoral students, though this was not universal. Where available, training built confidence, provided resources, and helped structure meaningful engagement. Given the vital role of PPI, students suggested such training should be embedded early in all doctoral research.

Ultimately, the integration of PPI strengthened the research and supported personal and professional growth for the doctoral students. All three doctoral researchers viewed public involvement as a genuine partnership, one that required time, humility, and sensitivity. Their reflections underscore that research is not only about generating data, but also about people, relationships, and carrying forward the stories entrusted to them.

### 2.3 The recommendations

Based on our collective experiences, whether completing a doctoral degree with strong PPI involvement, supporting doctoral students and PPI contributors, or coordinating PPI across multiple research projects, we have identified common challenges and practical recommendations. We have each seen, from different perspectives, how PPI can transform doctoral research:

- For students, early and meaningful engagement with PPI contributors can refine research questions, improve recruitment strategies and materials, and increase the relevance and impact of findings.
- For PPI contributors, being involved in doctoral projects offers the opportunity to shape the direction of research, share their lived experiences in meaningful ways, and build meaningful partnerships with emerging researchers. This collaboration also helps both contributors and researchers to understand issues more clearly than would be possible from an experiential or academic perspective alone.
- For coordinators, supporting doctoral students to embed PPI well can strengthen networks, enable sharing of good practice, and help ensure public voices are heard at the highest academic level, through involvement in doctoral research outputs, dissemination activities, and co-authored publications, and therefore, potentially, feed into policy-making.

From these perspectives, we have co-developed recommendations for each group. These are not rigid rules. They are guiding principles emerging from shared lessons that we believe can help make PPI in doctoral research more effective, inclusive, rewarding and successful for everyone involved.

Below, we have provided concise summary tables for each audience: Doctoral Students (see Table 1), PPI Contributors (see Table 2), and PPI Coordinators (see Table 3). These are designed for quick reference and to highlight the key priorities for successful collaboration. More detailed explanations, along with examples and practical tips, are provided in Appendices 1–3.

**Table 1:** PhD student recommendations

Section and Action Items	Further Detail
**1. Before You Start**	
1.1 Involve PPI contributors early	Start involving PPI contributors at the very beginning of your project (or as early as possible), ideally when you’re designing your research questions or intervention. This allows PPI contributors to shape the project’s direction, make it more relevant, and avoid missing important perspectives. Early involvement means they can help with priorities, design choices, or even recruitment strategies. It’s much harder (and may feel tokenistic) to add PPI later on.
1.2 Get support	If your university or institution has a PPI coordinator, work with them from the start. They can offer tailored advice, help you plan your approach, provide templates, and suggest practical steps for involving contributors meaningfully. If no coordinator exists, find mentors or colleagues with PPI experience. You can also look for PPI training or workshops around communication and inclusivity to build your skills before you start.
1.3 Understand PPI roles	Spend time learning about the range of ways PPI contributors can contribute. They might help set priorities, review participant materials for clarity and accessibility, advise on methods, help interpret results, or co-present findings. Knowing these roles in advance helps you invite contributors into activities that fit their interests and strengths, rather than limiting them to reviewing documents.
1.4 Plan for sustainability	Think beyond your first meeting. Develop a long-term engagement plan that outlines how you will maintain contact, share updates, and keep contributors meaningfully involved across all stages of your PhD. This includes considering funding for expenses, building flexibility into meeting schedules, and ensuring PPI contributors have opportunities to contribute to analysis, dissemination, and even post-project reflection.
**2. Building Relationships**	
2.1 Make a personal introduction	When meeting PPI contributors, clearly explain who you are, your background, your research area, and why this project is important to you. Avoid jumping into academic language or titles; make it personal and relatable. Sharing your own motivations builds trust and connection. This could help humanise the relationship.
2.2 Take time to build trust	Trust is not automatic; it grows through consistent, respectful, and transparent interactions. Doctoral researchers should recognise the importance of respect in every exchange, valuing contributors’ time, perspectives, and boundaries as equal to their own academic expertise. Get to know PPI contributors beyond their role as contributors. Ask about their interests, reasons for joining, and expectations. Create a welcoming space where people feel valued for who they are.
2.3 Be open and genuine	Be honest about what you know and do not know, what you can and cannot change, and about your own learning process. Showing vulnerability and openness encourages a safe environment for contributors to share freely, including constructive criticism or difficult experiences.
**3. Communication and Planning**	
3.1 Use accessible language	Adapt your language so it’s understandable to a broad audience. Avoid or explain jargon and technical terms and check regularly if everyone is following. Providing a glossary or visual aids can help. Remember: if people do not understand, they cannot give meaningful feedback.
3.2 Set clear expectations	Be upfront and outline exactly what you’re asking of PPI contributors. How many meetings? What types of tasks? How long is the expected commitment? Be upfront about time frames, roles, and any outputs. Also, clarify what they can expect from you. For example, updates, payments or reimbursements, and acknowledgements.
3.3 Discuss preferred terms	Some PPI contributors may dislike terms such as “patients,” “service users,” or “lay people.” Have an open conversation about what terms feel most comfortable and respectful. You might even offer a choice (e.g. “PPI contributor,” “expert by experience”) and use their preferences in all materials.
3.4 Plan for inclusion and accessibility	Work with contributors to determine the most effective communication channels (email, phone, online, or in-person) and tailor them to their individual needs. This could involve reimbursing travel costs, providing large-print materials, or ensuring meetings are scheduled at accessible times.
**4. During the Work**	
4.1 Show how feedback shapes the research	Always explain what you did with PPI input. Even if you cannot use every suggestion, clarify why and acknowledge the value of all feedback. Showing this feedback loop can encourage continued engagement and builds trust.
4.2 Be organised and respect time	Send agendas in advance, keep meetings on schedule, and share clear minutes or action points afterwards. Respect people’s time by not over-running and by avoiding last-minute changes unless absolutely necessary.
4.3 Be sensitive and offer support	If your topic involves sensitive or potentially upsetting content, discuss this upfront and prepare appropriate support (e.g. debrief time, signposting to support services). Check in regularly on how PPI contributors are feeling and be prepared to pause or adapt discussions if needed.
5.1 Discuss acknowledgements	Ask contributors how they would like to be acknowledged; this might include being named in papers, reports, or presentations, or even listed as co-authors. Some may prefer not to be publicly named, so always seek consent.
**5. Recognition and Thanks**	
5.2 Provide updates and feedback	Keep PPI contributors informed about project progress and how their contributions have influenced the outcomes. Update them on milestones, publications, and any changes. This helps them see the real impact of their involvement.
5.3 Clarify practical details	Be transparent about payment or reimbursement (e.g., hourly rates, travel expenses, potential tax/benefits implications), expected number of meetings, and any preparation work. Provide this information in writing so contributors can plan and make informed decisions, acknowledging that practical considerations around payment may vary for different contributors.

**Table 2:** PPI contributor recommendations

Section and Action Items	Further Detail
**1. Understanding your role**	
1.1 Recognise the value of your perspective	Your experiences and opinions can make research more meaningful and relevant. Do not feel you need specialist knowledge; your perspective is often what’s missing in academic discussions.
1.2 Understand time and commitment	Check in advance what time commitment is expected (e.g. number of meetings, reading documents). Ask about how long the project will last and what kind of activities you’ll be involved in, so you can decide if it fits for you.
1.3 Understand your influence	Your role can be more than just giving feedback. You might help shape the research question, suggest ways to make study materials clearer, point out barriers for potential participants, or advise on how findings are shared. This is genuine partnership work, not just consultation. Sometimes, not every idea can be taken forward, but talking together about what’s possible helps everyone stay on the same page.
**2. Building Relationships**	
2.1 Foster mutual respect	A good working relationship is built on trust, respect, and open communication. Acknowledge the pressures the student is under, just as they should acknowledge your time and commitment.
2.2 Share your motivations	Let the student know why you’re involved in the project. Understanding each other’s reasons for participating helps create a stronger and more empathetic working partnership.
2.3 Handle disagreements constructively	It’s natural to have different opinions at times. Approach disagreements with curiosity, asking “Can you explain why you’ve chosen that approach?” rather than making assumptions. This keeps the dialogue productive and respectful.
**3. Getting Involved**	
3.1 Ask questions freely	If something doesn’t make sense, ask for clarification. Your questions might help the student realise where they need to simplify or improve explanations. You should feel comfortable pausing discussions to ask for clearer language.
3.2 Give honest and constructive feedback	Be honest about what you think works or doesn’t. Constructive feedback helps improve research and makes it more practical. For example, instead of just saying “This is confusing,” you might say “I found this paragraph confusing because of the medical jargon, could it be rephrased in simpler terms?”. Share it kindly, criticism is most helpful when delivered constructively and respectfully.
3.3 Stay open to learning	You may encounter unfamiliar terms or new processes. Be open to learning alongside the researcher and do not feel pressured to understand everything straight away.
**4. Practicalities**	
4.1 Check logistics work for you	Make sure that meeting times suit your schedule, and that you’re comfortable with the technology or location. Ask for support if needed, it’s okay to request alternative ways of meeting or receiving information.
4.2 Look after your wellbeing	Some research topics may be emotionally challenging or triggering. Take breaks, ask for time out, or decide to step back temporarily or permanently if necessary. Your wellbeing should always come first.
4.3 Ask for updates	If you do not hear how your feedback was used, ask for updates. It’s reasonable to want to know what difference your contributions have made and to stay connected to the project’s progress.
**5. Ending your Involvement**	
5.1 Discuss stepping away	If you need to stop or reduce your involvement, discuss this with the student. There may be ways to stay involved at a reduced level, or you might suggest another person to take over your role.
5.2 Ask about impact	When your involvement finishes, ask for a summary of what happened, what the outcomes were, and how your feedback was used. This can help you feel closure and understand the value of your contribution.

**Table 3:** PPI coordinator recommendations

Section and Action Items	Further Detail
**1. Finding your Motivation**	
1.1 Keep patient and public voices central	As a coordinator, your role is more than administrative; you are a champion for ensuring that research reflects the needs and perspectives of the people it aims to serve. Every decision you make, from recruiting contributors to advising on study design, should be guided by the question: “Does this keep the patient and public voice at the heart of the project?”
1.2 Promote openness and accountability	Help create a culture where researchers are transparent about their decisions and receptive to feedback. This can mean encouraging students to share both successes and challenges with their PPI contributors, fostering trust and credibility.
1.3 Draw from your personal ‘why’	Whether you’re motivated by improving healthcare, empowering underrepresented groups, or advancing inclusive research practices, reconnecting with your personal motivation can sustain you through challenging moments and inspire those you work with.
**2. Coordinating Across Diverse Populations**	
2.1 Stay flexible	Doctoral projects vary widely in topic, pace, and scope, ranging from sensitive topics such as bereavement to technical ones like data science. Be ready to adjust your approach to fit the context, whether that means working with contributors who prefer informal conversations or facilitating highly structured workshops.
2.2 Reuse and adapt resources	Maintain a library of adaptable templates, guides, and checklists. This reduces duplication of effort, allowing students to get started more quickly. For example, a standard meeting agenda template can be easily tailored to a specific research project.
2.3 Work with empathy	Recognise that contributors bring lived experiences that may be emotionally charged, and that doctoral students may be navigating steep learning curves. A calm, empathetic approach helps maintain positive relationships across the board.
**3. Facilitating Collaboration**	
3.1 Encourage a learning mindset	Remind both students and contributors that meaningful involvement is a skill, not an instinct. Facilitation, plain language communication, and inclusive practices take time to develop, and that’s okay.
3.2 Champion plain English	Coach students on how to drop jargon and explain concepts clearly. Consider running short training sessions or providing glossaries that both students and PPI contributors can use.
3.3 Build long-term relationships	Where possible, match the same contributors with a project from start to finish. Continuity strengthens trust, deepens understanding, and reduces the time spent re-explaining background information.
3.4 Use creative methods	Not all contributors thrive in formal meeting settings. Introduce participatory approaches such as storytelling exercises, visual mapping, or interactive prototypes to gather richer insights.
3.5 Close the feedback loop	Ensure contributors always hear how their input was used, or why it wasn’t. This reinforces the value of their contributions and sustains motivation.
**4. Providing Resources and Training**	
4.1 Practical templates and tools	Offer resources such as plain-language consent form templates, meeting agendas, and PPI activity logs. Providing these ready-made tools saves time and helps consistency across projects.
4.2 Training opportunities	Coordinate workshops on facilitation, plain-language writing, and creative engagement methods. Consider offering joint training sessions where both PhD students and PPI contributors can learn together, fostering mutual understanding.
4.3 HRA-aligned guidance	Make sure all PPI activities align with Health Research Authority (HRA) principles, especially regarding ethics and meaningful involvement. This can help students avoid compliance issues later in the project.
**5. Strengthening PPI in PhD-Programmes**	
5.1 Ensure access to networks	Every PhD student should know who to contact for PPI support and have access to an established pool of contributors. Where networks do not exist, help students connect with relevant patient groups, charities, or community organisations.
5.2 Embed PPI in training	Advocate for PPI to be a core part of postgraduate training, not an optional extra. This helps create a research culture where involvement is seen as essential, not supplementary.
5.3 Secure sustainable funding	Work with departments to ensure that students have dedicated budgets for PPI expenses. This covers reimbursements, training, and materials, and avoids the common problem of projects running out of funds for involvement activities.
5.4 Engage committed supervisors	Encourage supervisors to understand PPI principles so they can support their students effectively. A well-informed supervisor can help keep PPI on track even during busy research phases.

### Strengths and limitations

A strength of this commentary is that it was co-developed by doctoral researchers, public contributors, and a PPI coordinator, ensuring that multiple perspectives shaped the recommendations. This collaborative approach helps to address concerns raised in previous literature about tokenism, as contributors were actively involved in the writing of the paper as well as the underlying doctoral projects.

At the same time, we recognise several limitations. Writing collaboratively across different roles involved negotiating language, perspectives, and priorities. There is no “one size fits all” approach to PPI in doctoral research. While we have aimed to provide practical recommendations, the experiences we share are inevitably shaped by our own disciplinary, institutional, and socio-cultural contexts. The language we chose to describe PPI may not resonate equally with all audiences, and we acknowledge that terms such as “contributors” or “patients” carry different meanings across settings. As this work was developed entirely from the perspectives of PPI contributors, doctoral students, and PPI coordinators, supervisors were not part of the recommendation development process; as a result, this paper is not intended as guidance for supervisors, and engaging supervisors in a future, dedicated piece of work would be a valuable next step.

Finally, this paper is presented as an honest, collective reflection. Capturing the richness of diverse doctoral experiences within a single commentary required us to balance depth with brevity, and some nuance may be lost. Nonetheless, we believe that presenting our collective insights offers a valuable starting point for further discussion and adaptation of PPI practices in doctoral research.

### Reflexivity

This work was co-produced by doctoral researchers with clinical backgrounds, public contributors with lived experience relevant to the research topics, and a public involvement coordinator with experience in patient engagement practice. The perspectives shared reflect these professional and experiential positions, which shaped how PPI was understood, discussed, and prioritised throughout the project. Decisions regarding the level of personal and demographic information reported were made collaboratively.

## Conclusion and future directions

In this commentary, we have shared reflections from doctoral researchers, public contributors, and a public involvement coordinator on what it can be like to embed PPI within doctoral health and social care research. Drawing on our collective experiences across different doctoral projects, we have explored the practical, relational, and emotional aspects of involvement, including the importance of early engagement, open communication, and supportive coordination. From these reflections, we offer a set of shared, experience-based recommendations intended to support others navigating PPI in doctoral research, recognising that these are not prescriptive or fixed, but adaptable to different contexts and needs.

In this paper, ‘long-term collaboration’ refers to continuity across the duration of a PhD, where repeated contact can support relationship-building despite limited resources. However, sustaining these relationships beyond the end of a doctorate may require wider programme- or institute-level infrastructure (e.g., doctoral training programmes/centres) or links with established groups [17].

Moving forward, the researchers have experienced or anticipate ongoing relationships with PPI contributors. This may involve further input and updates regarding ongoing pieces of work or direct involvement in new research projects. Such ongoing involvement may depend on the availability of ongoing funding for doctoral research to support PPI involvement. However, it is acknowledged that many PPI contributors chose to work voluntarily in research projects, including the PPI contributors in this author group.

## Data Availability

Not applicable.

## Appendices

### Appendix 1: PhD student recommendations checklist

**Table:**

Status (✓or χ)	Phase	Task Description
	Pre-Start	Involve Early: PPI contributors engaged during research question design.
	Pre-Start	Consultation: Met with university PPI coordinator or mentor.
	Pre-Start	Role Definition: Mapped out specific roles (reviewing, analysing, presenting).
	Initial Meeting	Personal Intro: Shared personal motivation and background (non-academic).
	Initial Meeting	Terminology: Agreed on preferred terms (e.g., “Contributor” vs. “Patient”).
	Planning	Accessibility: Communication channels and meeting times adjusted for contributors’ needs.
	Planning	Contributions: Written details provided regarding payments, expenses, and time expectations.
	Ongoing	Feedback Loop: Documented exactly how PPI input influenced the study after every meeting.
	Ongoing	Support: Debriefing or support services arranged for sensitive topics.
	Closing	Recognition: Consent obtained for naming contributors in papers/reports.

### Appendix 2: PPI contributor recommendations checklist

**Table:**

Status (✓or χ)	Phase	Task Description
	Pre-Start	Role clarity: I understand the expected time commitment and what I’ll be asked to do.
	Pre-Start	Influence: I’ve discussed how my input can shape the work (and that not all suggestions may be used).
	Initial Meeting	Motivation: I’ve shared why I want to be involved and what matters to me.
	Initial Meeting	Respect: We’ve agreed how we’ll communicate and handle differences of opinion.
	Planning	Questions welcomed: I feel able to ask for clarification and plain language at any point.
	Planning	Practicalities: Meetings/tech/access needs work for me (or I’ve asked for adjustments).
	Ongoing	Feedback: I give honest, constructive feedback and explain what could improve and why.
	Ongoing	Wellbeing: I know I can pause/step back if the topic is too difficult.
	Ongoing	Updates: I receive (or ask for) updates on how my input was used.
	Closing	Closure: I’ve requested a short summary of outcomes and the impact of my involvement.

### Appendix 3: PPI coordinator recommendations checklist

**Table:**

Status (✓or χ)	Phase	Task Description
	Pre-Start	Purpose: I’ve reinforced that PPI keeps public priorities central (not an “extra”).
	Pre-Start	Flexibility: I’ve adapted support to the project topic and contributors’ preferences.
	Pre-Start	Resources: Templates/tools are available (agenda, logs, plain-English examples).
	Early Set Up	Networks: Student knows how to access contributors and coordinator support.
	Planning	Training: Facilitation/plain-English/creative-methods training is signposted or offered.
	Ongoing	Continuity: Where possible, contributors are matched for start-to-finish involvement.
	Ongoing	Feedback loop: A clear “you said / we did (or why not)” approach is encouraged.
	Ongoing	HRA/ethics: PPI activities are consistent with HRA principles and guidance.
	Ongoing	Funding: Dedicated budget for reimbursement/training/materials is in place or pursued.
	Programme-Level	Embedding: PPI is advocated as part of doctoral training and culture (not optional).

### Appendix 4: Co-authors: group characteristics

**Table:**

Demographics	
**Sex**	
Male	3
Female	4
**Ethnicity**	
White British	7
**Age Range (years)**	30–80
***Role***	
PhD Student	3
PPI Contributor	3
PPI Coordinator	1
***Institute/Affiliation***	
University of Hull	5
University of St Andrews	2

### Appendix 5: PhD summaries

**Table:**

Student Initials	Summary
SPB	This PhD explored through multiple methodologies how people with multiple long-term conditions used and experienced healthcare towards the end of life. It involved a scoping review looking at how people with Advanced Multimorbidity were identified in research and clinical practice; a whole population analysis of people who had died with multiple long-term conditions and how this impacted their use of healthcare services towards the end of life; and a qualitative study of bereaved caregivers experiences of end of life care, using Interpretative Phenomenological Analysis.
AW	This PhD used constructivist grounded theory to explore how parentally bereaved children and their surviving parents experience and receive support. Through in-depth, co-produced interviews, the research worked closely with bereaved children, parents, and PPI partners to analyse and interpret their needs and identify what helps most. Categories and recommendations were co-constructed to improve support for both children and surviving parents following parental bereavement.
JC	This PhD used a mixed-methods, user-centred design approach to examine the feasibility, acceptability, and potential efficacy of an online intervention which delivered personalised exercise programming and lifestyle information to those living with and beyond lung cancer (called ExerciseGuide UK). Iterative patient and public involvement was conducted to co-develop the intervention website and supporting materials. PPI members were involved in the adaptations from usability testing to ensure the website was grounded in lived experience.

## Abbreviations

PPI: Patient and Public Involvement
NHS: National Health Service
HRA: Health Research Authority
NIHR: National Institute for Health and Care Research
